# Clinical Evaluation of Microendoscopy-Assisted Oblique Lateral Interbody Fusion

**DOI:** 10.3390/medicina57020135

**Published:** 2021-02-03

**Authors:** Tomohide Segawa, Hisashi Koga, Masahito Oshina, Katsuhiko Ishibashi, Yuichi Takano, Hiroki Iwai, Hirohiko Inanami

**Affiliations:** 1 Department of Orthopaedic Surgery, Inanami Spine and Joint Hospital, 3-17-5 Higashishinagawa, Shinagawa City, Tokyo 140-0002, Japan; masahito04031979@yahoo.co.jp (M.O.); luigi.igiul1030@gmail.com (Y.T.); h-iwai@iwai.com (H.I.); inanamihiro@gmail.com (H.I.); 2 Department of Orthopaedics, Iwai Orthopaedic Medical Hospital, 8-17-2 Minamikoiwa, Edogawa City, Tokyo 133-0056, Japan; hkoga0808@gmail.com (H.K.); nsd71255@gmail.com (K.I.); 3 Department of Neurosurgery, Iwai FESS Clinic, Suite 101, 8-18-4 Minamikoiwa, Edogawa City, Tokyo 133-0056, Japan

**Keywords:** oblique lateral interbody fusion (OLIF), lumbar lateral interbody fusion (LLIF), microendscope, minimally invasive surgery, extreme lateral interbody fusion (XLIF)

## Abstract

*Background and objectives*: Oblique Lateral Interbody Fusion (OLIF) is a widely performed, minimally invasive technique to achieve lumbar lateral interbody fusion. However, some complications can arise due to constraints posed by the limited surgical space and visual field. The purpose of this study was to assess the short-term postoperative clinical outcomes of microendoscopy-assisted OLIF (ME-OLIF) compared to conventional OLIF. *Materials and Methods*: We retrospectively investigated 75 consecutive patients who underwent OLIF or ME-OLIF. The age, sex, diagnosis, and number of fused levels were obtained from medical records. Operation time, estimated blood loss (EBL), and intraoperative complications were also collected. Operation time and EBL were only measured per level required for the lateral procedure, excluding the posterior fixation surgery. The primary outcome measure was assessed using the Japanese Orthopedic Association Back Pain Evaluation Questionnaire (JOABPEQ). The secondary outcome measure was assessed using the Oswestry Disability Index (ODI) and the European Quality of Life–5 Dimensions (EQ-5D), measured preoperatively and 1-year postoperatively. *Results*: This case series consisted of 14 patients in the OLIF group and 61 patients in the ME-OLIF group. There was no significant difference between the two groups in terms of the mean operative time and EBL (*p* = 0.90 and *p* = 0.50, respectively). The perioperative complication rate was 21.4% in the OLIF group and 21.3% in the ME-OLIF group (*p* = 0.99). In both groups, the postoperative JOABPEQ, EQ-5D, and ODI scores improved significantly (*p* < 0.001). *Conclusions*: Although there was no significant difference in clinical results between the two surgical methods, the results suggest that both are safe surgical methods and that microendoscopy-assisted OLIF could serve as a potential alternative to the conventional OLIF procedure.

## 1. Introduction

Numerous lumbar interbody fusion techniques are available for various spinal disorders [1]. Different techniques such as anterior lumbar interbody fusion (ALIF), posterior lumbar interbody fusion (PLIF), and transforaminal lumbar interbody fusion (TLIF) present a range of advantages and drawbacks [2,3,4,5,6,7]. ALIF allows for the restoration of intervertebral height by an enlargement of the foramen and spinal canal to achieve indirect posterior decompression [8,9,10]. In recent years, two major approaches of lumbar lateral interbody fusion (LLIF) have been popularized to offer more minimally invasive alternatives. One of these approaches is the extreme lateral interbody fusion (XLIF) procedure, which provides access to the lumbar spine using a true lateral approach [11]. Another recently introduced alternative to XLIF is the oblique lateral interbody fusion (OLIF) procedure that allows access to the lumbar spine and preservation of the psoas through the anterior oblique retroperitoneal approach. However, potential risks associated with LLIF can include injury to the iatrogenic lumbar plexus due to the dissected muscle fibers of the psoas, in addition to other complications caused by damage to critical structures near the vertebral body [12,13,14,15,16,17,18]. Harming the adjacent structures of the vertebral body can lead to fatal complications, including segmental arterial injury and bowel perforation. Moreover, these complications are compounded by the narrow surgical space and limited visual field of the procedure, as they are difficult to locate during surgery.

To address these complications, we have previously reported the advantages of microendoscopy-assisted XLIF over conventional XLIF [19]. In addition, the use of microendoscopy has been implemented to assist operative procedures since the introduction of OLIF at our hospital. The purpose of this study was to assess the short-term postoperative clinical outcomes of the microendoscopy-assisted OLIF compared to conventional OLIF and to clarify the advantages and disadvantages of this strategy.

## 2. Materials and Methods

We retrospectively investigated 75 consecutive patients who underwent OLIF or ME-OLIF at Inanami Spine and Joint Hospital from October 2016 to March 2019. Each surgeon who evaluated their surgical experience was board certified as an orthopaedic spine surgeon by the Japanese Society for Spine Surgery and Related Research (JSSR). Candidates were certified as a specialized spine surgeon by JSSR when they obtained experience of performing more than 300 spine surgeries as a primary operator.

Background information of the patients, including age, sex, diagnosis, and the number of fused levels, were obtained from medical records. Operation time, estimated blood loss (EBL), and intraoperative complications were also collected. Operation time and EBL were only measured per level required for the lateral procedure, excluding the posterior fixation surgery.

The primary outcome measure was assessed preoperatively and 1-year postoperatively using the Japanese Orthopedic Association Back Pain Evaluation Questionnaire (JOABPEQ) [20]. The JOABPEQ for assessing lower back pain contains 25 items with five subscales as an evaluation that is specific to the disease. These subscales include social function (four items), mental health (seven items), lumbar function (six items), walking ability (five items), and lower back pain (four items). Higher scores for each subscale, ranging from 0 to 100, denote better conditions. The secondary outcome measure was assessed using the Oswestry Disability Index (ODI; range from 0 to 100, with higher scores indicating more disability related to back pain) [21] and European Quality of Life–5 Dimensions (EQ-5D; range from 0 to 1, with higher scores indicating better quality of life) [21]. These two indices were measured preoperatively and 1-year postoperatively.

The mean values between the two groups were examined by the Shapiro–Wilk W-test to examine the normality of distribution. Statistical analysis was performed using the unpaired Students’ t-test to compare continuous variables between the two groups when the data followed normal distribution with homoscedasticity. Welch’s t-test was used for normally distributed data with heteroscedasticity. The Mann–Whitney U test was used if the data exhibited non-normal distribution. The Chi-square test or Fisher’s exact test was used to compare proportions. The effect size was assessed using Cohen’s d index, and the 1-β (power) was calculated. A *p*-value of <0.05 was considered statistically significant. Statistical analysis was performed using JMP 14 (SAS Institute, Cary, NC, USA).

All procedures performed in this study involving human participants adhered to the tenets of the 1964 Helsinki Declaration. In addition, procedures were in accordance with the ethical standards of the research committee of Iwai Medical Foundation (No. 20200507, 1 October 2019). Signed informed consent was obtained from all patients with disclaimer documents for the surgical procedure.

### Surgical Technique

The difference between ME-OLIF and conventional OLIF lies in whether or not a microendoscope is used. Therefore, the procedure for accessing the intervertebral disc is identical for both surgical procedures [22].

Patients were first placed in the right lateral decubitus position. Identification of the targeted intervertebral disc space was achieved with fluoroscopic guidance. At the center of the targeted segment, a 4-cm skin incision was made along fibers of the external oblique muscle. The internal and external oblique muscles and the transverse abdominal muscle were bluntly dissected in the direction of their fibers. Along the retroperitoneal fat tissue, blunt dissection was performed to access the retroperitoneal space. An anterior mobilization of the peritoneum provided exposure for the anatomical oblique lateral corridor. Finally, a retractor (OLIF25 Clydesdale Spinal System; Medtronic Sofamor Danek, Minneapolis, MN, USA) was placed.

**Figure 1:**
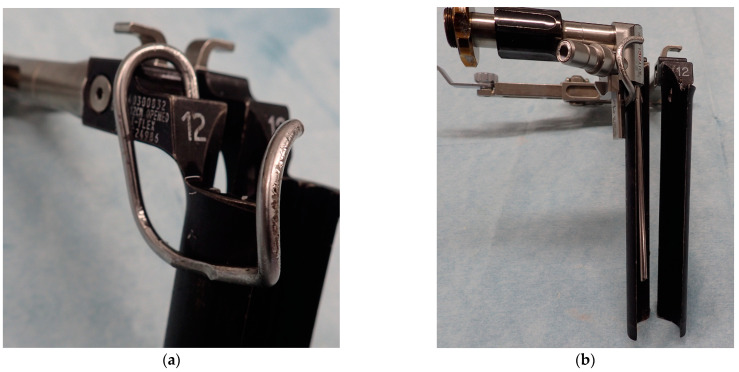
A customized attachment and intraoperative view. (**a**) A retractor with a custom-ordered attachment. (**b**) A microendoscope is attached inside a retractor.

**Figure 2:**
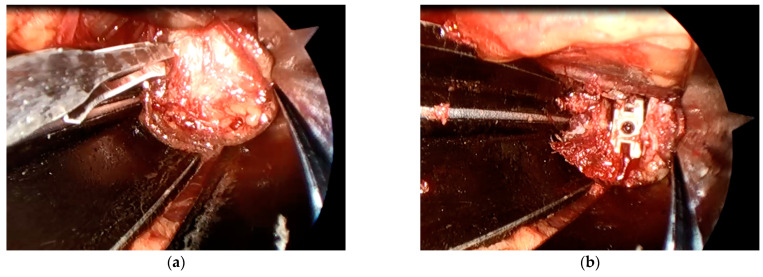
Intraoperative microendoscopic view of the retroperitoneal space. (**a**) The lateral part of the annulus fibrosis was clearly visualized, incised, and discectomized; (**b**) an interbody implant placement was performed.

A microendoscope (Medtronic Sofamor Danek, Memphis, TN, USA) was attached to a retractor with a customized attachment (Figure 1). Subsequently, the lateral part of the annulus fibrosis was clearly visualized, incised, and discectomized. Additionally, an interbody implant placement was performed (Figure 2). Following anterior fusion, patients were prone to undergo posterior fusion with pedicle screws via percutaneous procedures.

## 3. Results

Patient background characteristics for the 75 patients are shown in Table 1. This case series consisted of 14 patients in the OLIF group and 61 patients in the ME-OLIF group. The mean age at surgery was 66.4 years in the OLIF group and 64.5 years in the ME-OLIF group. All data were normally distributed and homoscedastic; thus, data were compared using Student’s t-test. There was no significant difference in patient background between the two groups.

The most common primary diagnosis was spondylolisthesis (OLIF: 8 patients, 57.1%; ME-OLIF: 38 patients, 62.3%), followed by degenerative disc disease (OLIF: 3 patients, 21.4%; ME-OLIF: 16 patients, 26.2%). Fifty-nine patients (OLIF: 11 patients, 78.6%; ME-OLIF: 48 patients, 78.7%) had a single vertebral level. Fourteen patients (OLIF: 3 patients, 21.4%; ME-OLIF: 13 patients, 21.3%) had two levels, and two patients in the ME-OLIF group had three levels.

The mean operative time per level required for the lateral procedure in the OLIF and ME-OLIF groups was 49.9 ± 14.1 min and 44.9 ± 12.7 min, respectively (*p* = 0.90). The mean EBL per level required for the lateral procedure in the OLIF group and ME-OLIF groups was 24.3 ± 27.9 mL and 24.3 ± 30.3 mL, respectively (*p* = 0.50). There was no significant difference between the groups in terms of the mean operative time per level and EBL per level.

Regarding intraoperative complications, three patients (21.4%) in the OLIF group and seven patients (11.5%) in the ME-OLIF group were clinically diagnosed with transient thigh pain/numbness. All patients recovered after three months of conservative treatment. Four patients (6.6%) in the ME-OLIF group had end-plate fractures. Two patients (3.3%) in the ME-OLIF group had segmental artery branches injury. There was no reoperation for these complications in the two groups.

The overall complication rate was 21.4% in the OLIF group and 21.3% in the ME-OLIF group. There was no significant difference between the groups in terms of the complication rate (*p* = 0.99).

Patient outcomes are shown in Table 2. All data exhibited normal distribution and are thus expressed as mean ± standard deviation. The postoperative JOABPEQ score improved significantly in both groups, but there was no significant difference in the rate of improvement between groups. The preoperative ODI score of 35.4 ± 8.2 in the OLIF group significantly improved postoperatively to 14.7 ± 12.8 (*p* < 0.001). The preoperative ODI score of 38.0 ± 13.3 in the ME-OLIF group improved significantly to 18.2 ± 14.5 (*p* < 0.001) postoperatively. There was no significant difference in the rate of improvement between groups. The preoperative EQ-5D score of 0.6 ± 0.1 in the OLIF group improved significantly to 0.8 ± 0.2 (*p* < 0.001) postoperatively. The preoperative EQ-5D score of 0.6 ± 0.2 in the ME-OLIF group improved significantly to 0.8 ± 0.2 (*p* < 0.001) postoperatively. There was no significant difference in the rate of improvement between groups.

## 4. Discussion

All primary and secondary outcomes improved significantly in both groups. There was no significant difference in the complication rate between groups. In addition, there were no visceral injuries such as ureteral and major vascular injury.

Some reports have described complications in OLIF surgery (Table 3) [23,24]. Abe et al. reviewed the incidence of perioperative complications in 155 patients who underwent OLIF surgery [23]. They reported that there were 75 complications (48.3%) during the intraoperative period. The most common complication was subsidence/endplate fracture (18.7%), followed mainly by transient thigh pain/numbness and/or psoas weakness (13.5%) and segmental artery injury (2.6%). Only three cases of permanent damage in ureter and nerves were reported. Fujibayashi et al. reviewed the complications in 1003 patients who underwent OLIF surgery and reported 153 complications (15.3%) [24]. Of these complications, 47.8% of cases occurred intraoperatively and 50.4% of cases were identified after the operation. The most common complication was sensory nerve injury (3.5%), followed mainly by transient thigh pain/numbness and/or psoas weakness (3.0%) and vertebral body fracture (2.6%). There was one major vascular injury.

In our study, the overall complication rate was 21.4% in the OLIF group and 21.3% in the ME-OLIF group. The most common complication in both groups was transient thigh pain/numbness 21.4% and 11.5%, respectively. Uribe et al. reported that a longer operative time could cause thigh pain transient thigh pain/numbness [25]. There was no significant difference in operative time between groups, but there was a tendency toward a shorter operative time in ME-OLIF group. This may affect the low incidence of thigh pain in the ME-OLIF group. End-plate fractures were observed in 6.6% of patients in the ME-OLIF group. Though there were no endplate fractures in OLIF group, all of these endplate fractures in ME-OLIF group occurred during the perioperative period. The reason might be that the cage was inserted with microendoscopic assistance. It may be influenced by the proficiency of the surgical procedure. Injuries to the segmental artery branches were observed in 3.3% of patients in the ME-OLIF group. Although our complication rate was similar to those previous reported, two patients exhibited injuries to the segmental artery branches in the ME-OLIF group. However, these complications were minor injuries to the vascular branches that could only be confirmed using a microendoscope. Though it may be handled by compression hemostasis during OLIF procedure, the ease of maintaining hemostasis was a notable advantage of microendoscopy-assisted surgery.

Microendoscopy-assisted spine surgery is widely practiced in Japan as a minimally invasive technique [26] and was first applied to lumbar disc herniation [27]. A more extended application of the technique has been described to perform spinal canal decompression and interbody fusion [28]. There are two major advantages for performing the microendoscopic technique. First, a microendoscopic lens is angled at 25° and the visual field of a microendoscope lies within the body during surgery. Therefore, better visualization of the lateral aspect is achieved compared to the unaided eye or surgical loupes, which are viewed from the exterior of the body (Figure 2). Secondly, the surgeon, assistant, and scrub nurse can simultaneously observe the same surgical field through the microendoscopic view. As a result, the progress of surgery can be accurately assessed in real time to enable a smooth and efficient workflow.

On the other hand, there are general disadvantages to microendoscopic surgery, such as a steep learning curve and increased complications. In particular, it is important to be proficient in bidimensional surgery using a microendoscope. However, there was no significant difference in both operative time and complication rate when comparing the two surgical procedures. There were no disadvantages to using a microendoscope, which was considered to be common in the early stages of starting ME-OLIF.

This study has two notable limitations. First, the sample size was too small to conclude the definitive efficacy of ME-OLIF. Although the effect size and 1-β (Table 1 and Table 2) of the data were found to be insufficient to detect significant differences, we believe that our findings would be particularly useful when combined with future studies in the context of a meta-analysis framework. Second, the long-term effect and safety of the procedure could not be properly evaluated due to the insufficient follow-up duration. However, we believe that future studies with an increased the study size will not yield an increased complication rate with the use of a microendoscope, as this technique is not a new concept but rather a modification of established methods. Therefore, we believe that ME-OLIF is a safe surgical procedure.

## 5. Conclusions

Although there was no significant difference in clinical results between the two surgical methods, the results suggest that both are safe surgical methods and that microendoscopy-assisted OLIF could serve as a potential alternative to the conventional OLIF procedure.

## Figures and Tables

**Table 1:** Patient background characteristics.

		OLIF	ME-OLIF	*p*-Value	Effect Size	(1-β)
N		14	61			
Sex						
	Females	9	35	0.64	0.14 †	0.16 †
	Males	5	26			
Age (years)		66.4 ± 8.4	64.5 ± 11.3	0.72	0.19 ‡	0.16 ‡
Diagnosis						
	Spondylolisthesis	8	38			
	Degenerative disc disease	3	16			
	Lumbar spinal stenosis	1	2			
	Lumbar foraminal stenosis	0	2			
	Degenerative scoliosis	1	1			
	Spondylolysis	0	2			
	Spondylosis deformans	1	0			
Number of fused levels						
	1	11	48			
	2	3	11			
	3	0	2			

‡ Assessed using Chi-square test; † assessed using Student’s *t*-test.

**Table 2:** Operative outcomes.

	Subscale		OLIF	ME-OLIF	*p*-Value	Effect Size	(1-β)
Operative time per level (min)			49.9 ± 14.1	44.9 ± 12.7	0.90	0.37	0.34
Blood loss per level (mL)			24.3 ± 27.9	24.3 ± 30.3	0.50	0.00	0.05
							
JOABPEQ score							
	Lower back pain	Pre	46.9 ± 29.9	45.6 ± 31.7	0.89	0.04	0.07
		Post	86.7 ± 27.7	76.7 ± 26.4	0.24	0.37	0.34
		Change	39.8	31.1	0.39		
		*p*-value	<0.001	<0.001			
	Lumbar function	Pre	50.6 ± 22.7	59.8 ± 26.5	0.20	0.37	0.35
		Post	76.7 ± 28.5	75.3 ± 25.7	0.86	0.05	0.07
		Change	26.1	15.4	0.15		
		*p*-value	<0.001	<0.001			
	Walking ability	Pre	50.0 ± 26.3	47.5 ± 31.7	0.76	0.09	0.09
		Post	86.2 ± 25.9	77.4 ± 31.2	0.28	0.3	0.27
		Change	36.2	29.9	0.54		
		*p*-value	<0.001	<0.001			
	Social life function	Pre	48.4 ± 14.6	43.4 ± 21.3	0.30	0.28	0.23
		Post	62.5 ± 27.8	64.6 ± 26.3	0.80	0.08	0.08
		Change	14.1	21.2	0.33		
		*p*-value	0.03	<0.001			
	Mental health	Pre	50.1 ± 18.3	50.7 ± 17.7	0.91	0.03	0.06
		Post	66.6 ± 22.3	62.6 ± 17.6	0.54	0.2	0.16
		Change	16.5	11.9	0.33		
		*p*-value	<0.001	<0.001			
ODI score		Pre	35.4 ± 8.2	38.0 ± 13.3	0.35 †	0.24 †	0.2
		Post	14.7 ± 12.8	18.2 ± 14.5	0.38 †	0.26 †	0.21
		Change	−20.7	−19.8	0.86 ‡		
		*p*-value	<0.001 *	<0.001 *			
EQ5D score		Pre	0.6 ± 0.1	0.6 ± 0.2			
		Post	0.8 ± 0.2	0.8 ± 0.2	0.30		
		Change	0.27	0.20	0.57		
		*p*-value	<0.001	<0.001			

* For those applicable, changes in each group before and 1 year after surgery were assessed using a paired *t*-test. † Welch’s *t*-test was used to compare the mean values between the two groups. ‡ the Mann–Whitney U test was used to compare mean pre- and postoperative 1-year differences for each group.

**Table 3:** Complications reported in the literature.

Author	Patients	Complications Reported (%)
Abe et al. [23].	155	Subsidence/endplate fracture (18.7)
		Transient thigh pain/numbness and/or psoas weakness (13.5)
		Segmental artery injury (2.6), ureter and nerves injury (1.9)
Fujibayashi et al. [24].	1003	Sensory nerve injury (3.5)
		Transient thigh pain/numbness and/or psoas weakness (3.0)
		Vertebral body fracture (2.6), major vascular injury (0.1)

## Data Availability

The datasets generated and analyzed during the current study are available from the corresponding author upon reasonable request.

## References

[1] Fraser R.D. (1995). Interbody, posterior, and combined lumbar fusions. Spine.

[2] Cloward R.B. (1985). Posterior lumbar interbody fusion updated. Clin. Orthop. Relat. Res..

[3] Penta M., Fraser R.D. (1997). Anterior lumbar interbody fusion. A minimum 10-year follow-up. Spine.

[4] Harms J.G., Jeszenszky D. (1998). Die posteriore, lumbale, interkorporelle Fusion in unilateraler transforaminaler Technik. Oper. Orthop. Traumatol..

[5] Kang B.U., Choi W.C., Lee S.H., Jeon S.H., Park J.D., Maeng D.H., Choi Y.G. (2009). An analysis of general surgery-related complications in a series of 412 minilaparotomic anterior lumbosacral procedures. J. Neurosurg. Spine.

[6] Brantigan J.W., Steffee A.D., Lewis M.L., Quinn L.M., Persenaire J.M. (2000). Lumbar interbody fusion using the Brantigan I/F cage for posterior lumbar interbody fusion and the variable pedicle screw placement system: Two-year results from a Food and Drug Administration investigational device exemption clinical trial. Spine.

[7] Christensen F.B., Hansen E.S., Eiskjaer S.P., Høy K., Helmig P., Neumann P., Niedermann B., Bünger C.E. (2002). Circumferential lumbar spinal fusion with Brantigan cage versus posterolateral fusion with titanium Cotrel-Dubousset instrumentation: A prospective, randomized clinical study of 146 patients. Spine.

[8] Castellvi A.E., Nienke T.W., Marulanda G.A., Murtagh R.D., Santoni B.G. (2014). Indirect decompression of lumbar stenosis with transpsoas interbody cages and percutaneous posterior instrumentation. Clin. Orthop. Rel. Res..

[9] Kepler C.K., Sharma A.K., Huang R.C., Murtagh R.D., Santoni B.G. (2012). Indirect foraminal decompression after lateral transpsoas interbody fusion. J. Neurosurg. Spine.

[10] Takahashi K., Kitahara H., Yamagata M., Murakami M., Takata K., Miyamoto K., Mimura M., Akahashi Y., Moriya H. (1990). Long-term results of anterior interbody fusion for treatment of degenerative spondylolisthesis. Spine.

[11] Ozgur B.M., Aryan H.E., Pimenta L., Taylor W.R. (2006). Extreme lateral interbody fusion (XLIF): A novel surgical technique for anterior lumbar interbody fusion. Spine J..

[12] Guérin P., Obeid I., Gille O., Bourghli A., Luc S., Pointillart V., Cursolle J.C., Vital J.M. (2011). Safe working zones using the minimally invasive lateral retroperitoneal transpsoas approach: A morphometric study. Surg. Radiol. Anat..

[13] Sofianos D.A., Briseño M.R., Abrams J., Patel A.A. (2012). Complications of the lateral transpsoas approach for lumbar interbody arthrodesis. A case series and literature review. Clin. Orthop. Relat. Res..

[14] Knight R.Q., Schwaegler P., Hanscom D., Roh J. (2009). Direct lateral lumbar interbody fusion for degenerative conditions. Early complication profile. J. Spinal Disord. Tech..

[15] Moro T., Kikuchi S., Konno S., Yaginuma H. (2003). An anatomic study of the lumbar plexus with respect to retroperitoneal endoscopic surgery. Spine.

[16] Brau S.A. (2002). Mini-open approach to the spine for anterior lumbar interbody fusion: Description of the procedure, results and complications. Spine J..

[17] Rodgers W.B., Gerber E.J., Patterson J. (2011). Intraoperative and early postoperative complications in extreme lateral interbody fusion. An analysis of 600 cases. Spine.

[18] Ahmadian A., Deukmedjian A.R., Abel N., Dakwar E., Uribe J.S. (2013). Analysis of lumbar plexopathies and nerve injury after lateral retroperitoneal transpsoas approach: Diagnostic standardization. A review. J. Neurosurg. Spine.

[19] Segawa T., Koga H., Inanami H. (2017). Clinical evaluation of microendoscopy-assisted extreme lateral interbody fusion. J. Spine Surg..

[20] Fukui M., Chiba K., Kawakami M., Kikuchi S., Konno S., Miyamoto M., Seichi A., Shimamura T., Shirado O., Taguchi T. (2009). Subcommittee of the Clinical Outcome Committee of the Japanese Orthopaedic Association on Low Back Pain and Cervical Myelopathy Evaluation. JOA Back Pain Evaluation Questionnaire (JOABPEQ)/JOA Cervical Myelopathy Evaluation Questionnaire (JOACMEQ) The report on the development of revised versions April 16, 2007. J. Orthop. Sci..

[21] Strömqvist B., Fritzell P., Hägg O., Jönsson B., Swedish Society of Spinal Surgeons (2009). The Swedish Spine Register: Development, design and utility. Eur. Spine J..

[22] Silvestre C., Mac-Thiong J.-M., Hilmi R., Roussouly P. (2012). Complications and morbidities of mini-open anterior retroperitoneal lumbar interbody fusion: Oblique lumbar interbody fusion in 179 patients. Asian Spine J..

[23] Abe K., Orita S., Mannoji C., Motegi H., Aramomi M., Ishikawa T., Kotani T., Akazawa T., Morinaga T., Fujiyoshi T. (2017). Perioperative Complications in 155 Patients Who Underwent Oblique Lateral Interbody Fusion Surgery: Perspectives and Indications from a Retrospective, Multicenter Survey. Spine.

[24] Fujibayashi S., Kawakami N., Asazuma T., Ito M., Mizutani J., Nagashima H., Nakamura M., Sairyo K., Takemasa R., Iwasaki M. (2017). Complications Associated with Lateral Interbody Fusion: Nationwide Survey of 2998 Cases During the First 2 Years of Its Use in Japan. Spine.

[25] Uribe J.S., Isaacs R.E., Youssef J.A. (2015). Can triggered electromyography monitoring throughout retraction predict postoperative symptomatic neuropraxia after XLIF? Results from a prospective multicenter trial. Eur. Spine J..

[26] Yoshimoto M., Iwase T., Takebayashi T., Ida K., Yamashita T. (2014). Microendoscopic discectomy for far lateral lumbar disk herniation: Less surgical invasiveness and minimum 2-year follow-up results. J. Spinal Disord. Tech..

[27] Foley K.T., Smith M.M. (1997). Microendoscopic discectomy. Tech. Neurosurg..

[28] Isaacs R.E., Podichetty V.K., Santiago P., Sandhu F.A., Spears J., Kelly K., Rice L., Fessler R.G. (2005). Minimally invasive microendoscopy-assisted transforaminal lumbar interbody fusion with instrumentation. J. Neurosurg. Spine.

